# Investigation of Relationship between Hepatitis B Virus and Gastric Adenocarcinoma

**Published:** 2012-07-30

**Authors:** M Ghasemi, L Vahedi Larijani, S Abediankenari

**Affiliations:** 1 Department of Pathology, Faculty of Medicine, Mazandaran University of Medical Sciences, Sari, Iran; 2 Department of Microbiology and Immunology, Faculty of Medicine, Mazandaran University of Medical Sciences, Sari, Iran

**Keywords:** HBV, Gastric cancer, Adenocarcinoma

Dear Editor,

Gastric cancer is the second leading cause of cancer-related deaths worldwide [1][2][3] while some genetic and environmental factors such as infectious agents were shown to be linked to carcinogenesis.[2] Infectious agents notably viruses were shown to induce 20-25% of all cancers around the globe.[4][5] Helicobacter pylori (H. pylori) is one of those infectious agents to be related to gastric carcinogenesis.[1][2] Epstein bar virus was inconstantly demonstrated to be associated with gastric cancer that may be due to the presence of the cofactor of H. pylori that can immortalize abnormal epithelial cells.[6]

Hepadnaviridae, the most powerful infectious related cause of hepatoma has been reported with other malignancies such as renal cell carcinoma, gastric and oral cancers.[7][8][9] One study showed an association between anti-hepatitis C virus (HCV) antibody in 10.6% of sera of patients and gastric cancer.[10] Gastric cancer is assumed to be more prevalent in cirrhotic patients.[9] Expression of hepatitis B virus (HBV) antigens in H. pylori bearing gastric mucosa was shown by immunostaining methods.[11] Here we evaluated the association between HBV infection and gastric carcinoma in north of Iran.

We collected one hundred formalin-fixed paraffin embedded blocks of biopsies confirmed as gastric cancer in pathology department of our university belonging to patients who underwent gastrectomy during 2005-2010. Besides, 100 blocks of normal gastric mucosa tissues were provided as control group. Polymerase chain reaction for HBV DNA detection was performed.

Sixty nine percent of gastric cancer patients were male (Male/female ratio: 69/31) with a mean age of 67.14±10.98 years. The most common histological type of adenocarcinoma was intestinal type (68%) followed by diffuse (26%) and mixed (7%) types while the location was respectively proximal (57%), distal (22%) and diffuse (6%). The majority patients were diagnosed at advanced stage III (68%) followed by stage 2 (19%), stage 4 (9%) and stage 1 (4%). The most common grade was grade 2 (52%), grade 1 (41%) and grade 3 (7%).

**Fig. 1:**
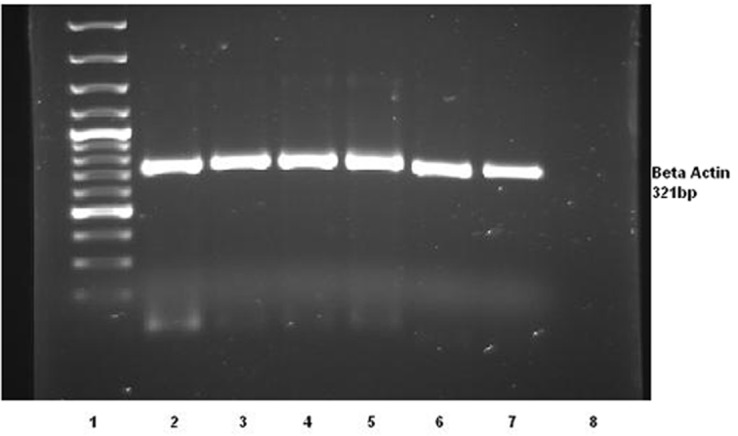
Agarose gel electrophoresis illustrated a positive control for patients DNA (Beta actin as house keeping gene) compared to negative gastric cancer cases. 1: DNA ladder (50 bp-1000bp), 2, 3, 4, 5, 6, 7: Beta actin (321 bp), 8: Negative con-trol.

HBV genome was undetectable in all cases and control subjects using PCR (Figure 1).

Previous studies confirmed the role of HBV in the pathogenesis of hepatocellular carcinoma.[4][5] Besides, limited studies have revealed association between HBV antigens and/or antibodies with other malignancies such as renal carcinoma, oral cancer, and gastric carcinoma.[7][8][9][10] Zullo et al. showed 2.6 folds increases in prevalence of gastric cancer in cirrhotic patients compared to non-cirrhotic individuals using endoscopic and histological examinations.[9]

Chen et al. showed the expression of HBV antigens in H. pylori infected gastric mucosa of patients with chronic liver disease using immunostaining.[11] Our findings showed that HBV genome was undetectable in gastric cancers. We demonstrated that HBV was not correlated with gastric carcinoma in north of Iran.
